# Two-in-One Hybrid Sensor Based on PV4D4/AgAu/TiO_2_ Structure for Carbon Dioxide and Hydrogen Gas Detection in Biomedical and Industrial Fields

**DOI:** 10.3390/bios16010005

**Published:** 2025-12-22

**Authors:** Mihai Brinza, Lynn Schwäke, Stefan Schröder, Cristian Lupan, Nicolai Ababii, Nicolae Magariu, Maxim Chiriac, Franz Faupel, Alexander Vahl, Oleg Lupan

**Affiliations:** 1 Center for Nanotechnology and Nanosensors, Department of Microelectronics and Biomedical Engineering, Technical University of Moldova, 168 Stefan cel Mare Av., MD-2004 Chisinau, Moldova; mihai.brinza@mib.utm.md (M.B.); lysc@tf.uni-kiel.de (L.S.); cristian.lupan@mib.utm.md (C.L.); nicolai.ababii@fcim.utm.md (N.A.); nicolae.magariu@mib.utm.md (N.M.); maxim.chiriac1@mib.utm.md (M.C.); 2 Department of Materials Science, Chair for Multicomponent Materials, Kiel University, Kaiserstraße 2, D-24143 Kiel, Germany; alexander.vahl@inp-greifswald.de; 3 Chair for Composite Materials, Faculty of Engineering, Kiel University, Kaiserstraße 2, D-24143 Kiel, Germany; 4 Leibniz Institute for Plasma Science and Technology, Felix-Hausdorff-Str. 2, 17489 Greifswald, Germany; 5 Functional Nanomaterials, Faculty of Engineering, Institute for Materials Science, Kiel University, Kaiserstra2, 24143 Kiel, Germany

**Keywords:** polymer coating, PV4D4, TiO_2_, hydrogen, carbon dioxide, medical applications, thermal annealing

## Abstract

A novel two-in-one sensor for both carbon dioxide and hydrogen detection has been obtained based on a hybrid heterostructure. It consists of a 30 nm thick TiO_2_ nanocrystalline film grown by atomic layer deposition (ALD), thermally annealed at 610 °C, and subsequently coated with bimetallic AgAu nanoparticles and covered with a PV4D4 nanolayer, which was thermally treated at 430 °C. Two types of gas response behaviors have been registered, as *n*-type for hydrogen gas and *p*-type semiconductor behavior for carbon dioxide gas detection. The highest response for carbon dioxide has been registered at an operating temperature of 150 °C with a value of 130%, while the highest response for hydrogen gas was registered at 350 °C with a value of 230%, although it also attained a relatively good gas selectivity at 150 °C. It is considered that a thermal annealing temperature of 610 °C is better for the properties of TiO_2_ nanofilms, since it enhances gas sensor sensitivity too. Polymer coating on top is also believed to contribute to a higher influence on selectivity of the sensor structure. Accordingly, to our previous research where PV4D4 has been annealed at 450 °C, in this research paper, a lower temperature of 430 °C for annealing has been used, and thus another ratio of cyclocages and cyclorings has been obtained. Knowing that the polymer acts like a sieve atop the sensor structure, in this study it offers increased selectivity and sensitivity towards carbon dioxide gas detection, as well as maintaining a relatively increased selectivity for hydrogen gas detection, which works as expected with Ag and Au bimetallic nanoparticles on the surface of the sensing structure. The results obtained are highly important for biomedical and environmental applications, as well as for further development of the sensor industry, considering the high potential of two-in-one sensors. A carbon dioxide detector could be used for assessing respiratory markers in patients and monitoring the quality of the environment, while hydrogen could be used for both monitoring lactose intolerance and concentrations in cases of therapeutic gas, as well as monitoring the safe handling of various concentrations.

## 1. Introduction

As the global population continues to grow, so does its demand for energy, transportation and consumables, leading to a significant increase in air pollution and environmental degradation [1,2]. These factors lead to global environmental challenges, which not only affect ecosystems in general but also have a specific impact on human health and behavior [3,4]. To maintain air quality and control emissions effectively, there is a need for systems capable of accurate gas and pollutant detection [1,5,6,7]. To meet these growing demands, gas sensor research increasingly focuses on the interaction of nanostructures with gases and volatile organic compounds (VOCs), as nanostructures exhibit unique properties due to their nanometric dimensions [8], which makes them highly suitable for sensitive gas sensing devices. In particular, the detection of carbon dioxide (CO_2_) and hydrogen (H_2_) gases is attracting substantial interest, not only from the scientific community, but also from various industrial fields, including energy, health care and medicine.

Carbon dioxide is a compound that plays a crucial role in a wide range of applications, making its monitoring an important aspect in various areas such as indoor and outdoor air quality monitoring [9,10], fire detection [11] or detection of incipient food spoilage [12,13]. Furthermore, carbon dioxide raises interest even in electrical vehicle safety, as a component found during battery vent-gas thermal runaway [14,15]. In the medical field and social assistance industry, the use of CO_2_ is also well-established [16,17,18]. It is, for example, used as an indicator for the respiratory and circulatory system in the human body, enabling the efficacy assessment of thrombolytic therapy [19] and even a footprint of anesthesia after different types of surgical operations [20]. However, blood gases such as CO_2_ are usually measured through blood sampling, which not only requires trained personnel but also poses an infection risk for the patient. Hence, development of a non-invasive monitoring technique, e.g., gas sensors, enabling the analysis of exhaled breath, has been a point of interest for several years, if not decades [16,21]. These interests are further amplified by global population growth and the associated health risks, as well as by recent pandemic crises such as the COVID-19 pandemic.

However, accurately monitoring carbon dioxide remains challenging. This has intensified interest in more complex metal oxide systems, such as lanthanum-containing structures [21,22], metal–TiO_2_–metal meta surfaces [23] and polymer-coated fiber Bragg gratings [22]. Furthermore, methods for CO_2_ detection using different sensors with liquid or paste-like electrolytes have been developed [24].

Hydrogen gas is widely recognized as a clean energy carrier [13,25]. Due to its high potential as an alternative to fossil fuels [26,27], hydrogen plays an active role in the automotive industry as fuel for electric cells [28], and is also used as fuel in space applications [29]. As such, effective hydrogen detection and monitoring systems are essential for the safe handling and application of hydrogen [13,26,30,31]. Beyond its energy-related applications, hydrogen has found use in various fields, such as the food industry. Here, hydrogen has been researched as a method for reducing natural senescence and microbial spoilage, thereby extending the shelf life of different products [32,33]. On the other hand, hydrogen’s potential has also been studied in the medical field [31]. A recent study investigated H_2_ as a potential biomarker for inflammatory bowel disease [34]. Other studies have focused on breath tests that link hydrogen with gastrointestinal disorders [35,36], while excessive levels are associated with lactose intolerance [37]. In recent years, hydrogen has been studied even as a preventive and therapeutic medical gas [38], improving the survival rate in zymosan-induced inflammation [39], as a means of alleviating sepsis-induced neuro-inflammation and cognitive impairment [40] and as an antioxidant and anti-inflammatory agent for stress-related diseases [41]. Thus, although there are numerous hydrogen detectors [27], the wide range of application demands constant improvement and further development of new sensors [10].

To address these demands, various sensors have been proposed in recent studies [7,13,26,27]. Different concepts, such as Pd-decorated oxides [10,42], hybrid structures with Au nanoparticles [43,44] or both Pt and Au [45], have been investigated to improve gas sensing properties. In previous studies, we used Au nanoparticles on zinc oxide [44,46], Ag and Pt nanoparticles on titanium oxide [47], polymer coating of PV4D4 [47,48,49] and even a copolymer P(V3D3 + TFE) [50] structure based on PV3D3 [51] and PTFE [52,53] for improving selectivity and sensitivity. Polymer use on top of the sensor structure is also intended to solidify humidity resistance, as it is known that the response of TiO_2_ structures diminishes at certain relative humidity levels [54].

This study proposes a novel, two-in-one gas sensing device capable of detecting carbon dioxide and hydrogen gas, targeting applications in both the medical and industrial fields. To evaluate its gas sensing properties, similar devices have been tested with several gases and at different operating temperatures. The influence of polymer coating on top of the sensor and its structural changes through thermal annealing has been assessed by both FTIR analysis and experimental data.

## 2. Materials and Methods

### 2.1. Sample Fabrication

**Figure 1:**
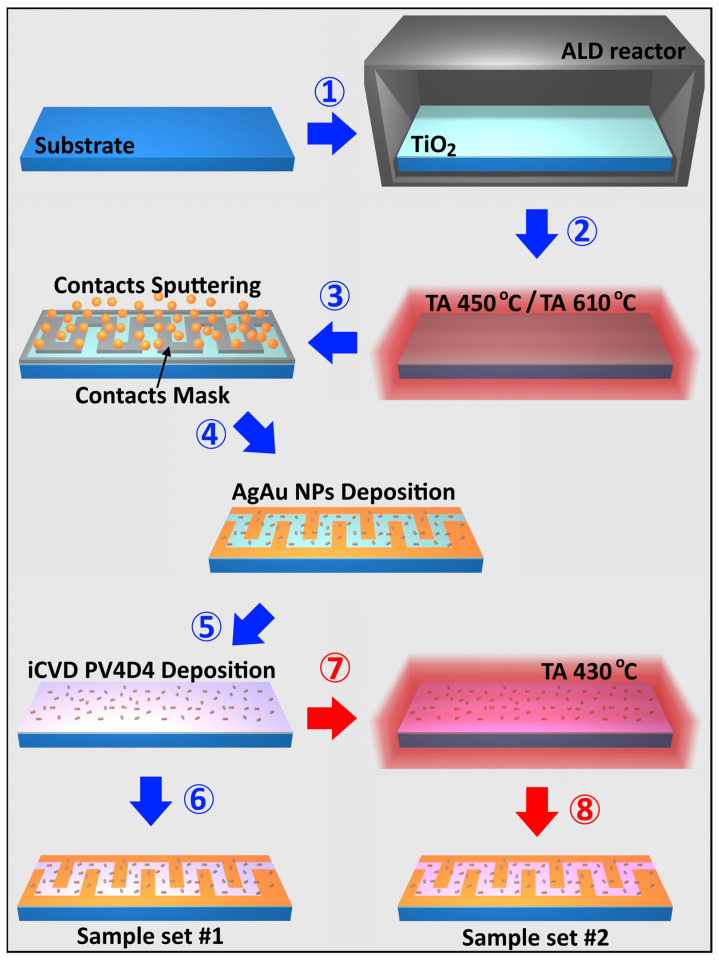
Fabrication steps for obtaining TiO_2_-based structures coated with AgAu nanoparticles and PV4D4 films.

This paper presents gas sensing structures consisting of TiO_2_ nanolayers that underwent different thermal annealing regimes (450 °C, 610 °C). These nanolayers were subsequently covered with nanodots and coated with a polymeric film. The sample sets were obtained according to the fabrication steps described in Figure 1.

The fabrication process is first prepared by cleaning the glass substrates (microscope glass slides of dimensions 76 mm × 25 mm × 1 mm, Thermo Scientific) with acetone, ethanol and a paper towel. TiO_2_ layers with a thickness of 30 nm were grown by atomic layer deposition (ALD), in a Picosun R-200 series reactor (Figure 1, *step 1*) [55]. Subsequently, the obtained samples were thermally processed at a temperature of 450 °C (Figure 1, *step 2*), after which Au contacts were sputtered on top of the structures through a meander-shaped mask (Figure 1, *step 3*). AgAu nanoparticles, in an atomic ratio of approximately 25% silver and 75% gold, as this ratio showed the best results for gas sensor tuning in previous papers [43,45,56,57], were deposited on the obtained surface using a specialized high-vacuum deposition system equipped with a custom-designed Haberland-type Gas Aggregation Source (GAS) with a custom multicomponent target, as reported in one of our previous works (Figure 1, *step 4*) [57]. SEM images can be observed in a previous publication, where the precision of manufacturing TiO_2_:Au has been proven [55]. The obtained sensors were coated with approximately 25 nm poly(1,3,5,7-tetravinyl-1,3,5,7-tetramethylcyclotetrasiloxane) (PV4D4) polymer via initiated chemical vapor deposition (iCVD) using a custom-built reactor (Figure 1, *step 5*), resulting in sample set #1, which was analyzed without further heat treatment (Figure 1, *step 6*). Sample set #2, on the other hand, was obtained by thermal heating PV4D4-coated sensors to 430 °C (Figure 1, *step 7*, TA) to produce heat-treated samples, TA, for further analysis (Figure 1, *step 8*).

### 2.2. Sample Characterization

For chemical analysis, Fourier-transform infrared spectroscopy (FTIR) was performed on approximately 400 nm thick reference samples. FTIR spectra were acquired using a Bruker INVENIO R spectrometer, (Bruker Optik GmbH, Ettlingen, Germany). The measurements covered a wavenumber range of 7500 to 370 cm^−1^, with 32 scans recorded at a resolution of 4 cm^−1^. For detailed analysis, a narrower range of 4000 to 700 cm^−1^ was selected.

The recorded FTIR data were processed using Origin (OriginLab 2024), where baseline correction (spline method), atmospheric compensation (CO_2_), normalization and smoothing (Savitzky–Golay method) were applied.

The gas sensing properties of the two sets of samples were calculated accordingly for the two types of response. As is well-known, electrical charge carriers in semiconductors can be of two types: holes and electrons. Therefore, two types of response have been registered: *p*-type for sensors where electrical resistance is rising and electrical current decreases to lower levels, and vice versa for *n*-type behavior, also mentioned in a previous work [58]. For the *n*-type response, formulas from a previous article have been used [48].

For *p*-type behavior, calculations are represented in Equation (1), where gas response *S* has been determined using the ratio of electrical resistances in air (*R_air_*) and during gas exposure (*R_gas_*), respectively.

(1) $$S=\frac{R_{gas}-R_{air}}{R_{air}}\times 100\%$$

The gas response of the developed samples was measured by using a custom-built setup and protocol, based on a computer-controlled source meter (Keithley 2400, Keithley, Cleveland, OH, USA), as previously described [59]. During measurements, the samples were heated to different operating temperatures (OPTs), while gases such as hydrogen, n-butanol, 2-propanol, ethanol, acetone, ammonia, carbon dioxide and methane were introduced into the chamber to maintain a gas concentration of 100 ppm.

## 3. Results and Discussion

### 3.1. FTIR Characterization

**Figure 2:**
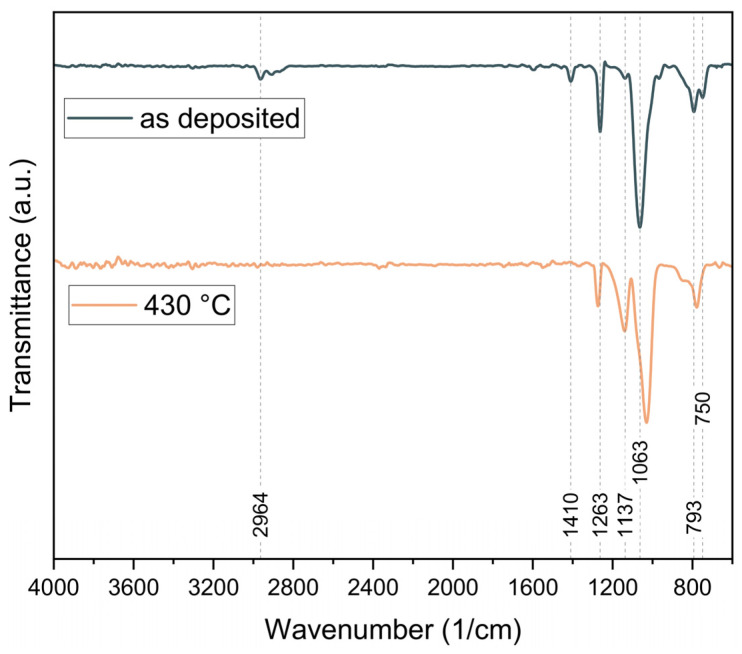
FTIR spectrum of the as deposited PV4D4 thin film and after thermal annealing at 430 °C.

As shown in Figure 2, the FTIR spectrum of the as-deposited PV4D4 film indicates successful polymerization, as there are no significant bands above 3000 cm^−1^, associated with unreacted vinyl groups. The formation of the polymer backbone through reacted vinyl groups contributes to the C-H stretching band at 2964 cm^−1^ and lower, associated with sp^3^-hybridized carbon atoms.

After thermal annealing at 430 °C, the C-H-associated bands in the FTIR spectrum nearly vanish, indicating a change in the polymer’s structure. This is further supported by the shift in the siloxane ring-associated band to a lower wavenumber (1030 cm^−1^), as well as by the appearance of a pronounced band at 1137 cm^−1^.

Additionally, the preservation of the tetrasiloxane ring structure is confirmed by the dominant Si–O–Si asymmetric stretching band at 1063 cm^−1^. Further bands at 750 cm^−1^, 1263 cm^−1^ and 1410 cm^−1^ can be attributed to Si–CH_3_ modes, including rocking, bending and deformation of the methyl groups [60].

Additionally, the emergence of a band at 779 cm^−1^, in combination with the reduction in the asymmetric Si-C stretching (at 750 cm^−1^) and methyl rocking band (793 cm^−1^) suggests, according to Trujillo et al. [61], the formation of O_3_Si(CH_3_) environments. These spectral changes are consistent with the literature and indicate the conversion of the polymer’s initial siloxane ring structure into a silsesquioxane-cage-like structure, due to thermal treatment [61].

**Figure 3:**
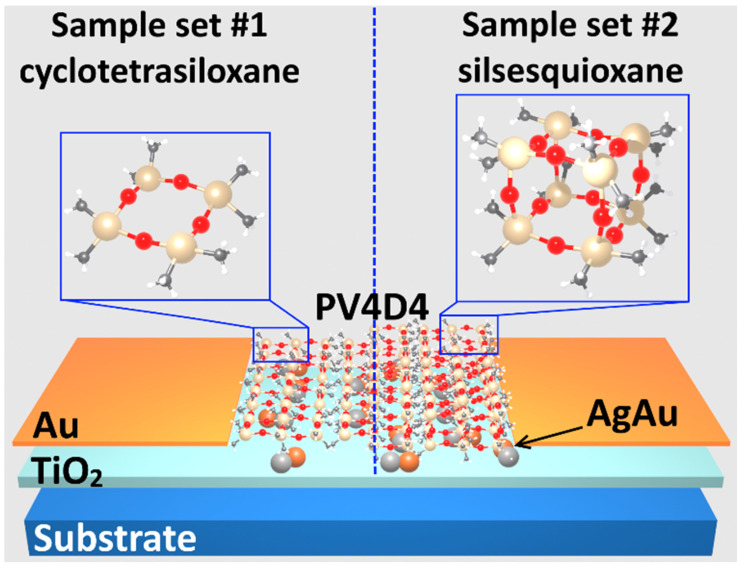
Samples representation in cross-section of TiO_2_ structures coated with PV4D4 before (cyclotetrasiloxane) and after (silsesquioxane) heat treatment.

In Figure 3 we propose a conceptual design for the visualization of the obtained samples in a cross-section. As observed, in the first sample set #1, the coated polymer has a cyclo structure, while the second sample set #2 is in cage structure, as a result of thermal annealing at 430 °C.

### 3.2. Sensory Properties

The gas response of the sample was measured using a custom setup and protocol based on a computer-controlled source-meter (Keithley 2400) described previously, including more detailed parameters such as flow, concentrations and equations for obtaining the performances of the specimens [47,50,59,62].

**Figure 4:**
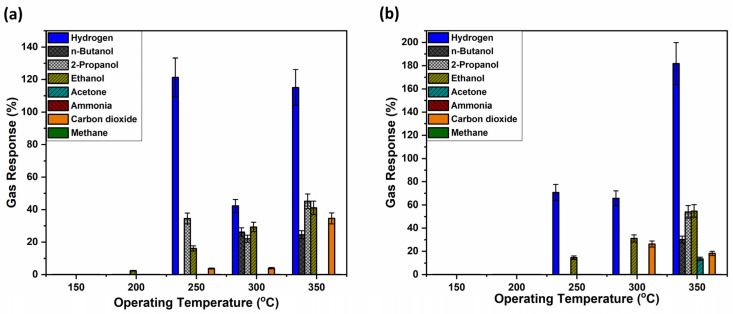
Gas response of the hybrid sensors based on TiO_2_ layers thermally annealed at 450 °C, covered with AgAu nanoparticles and polymers measured for different gases and operation temperatures with thermally treated and untreated polymer coatings on top. Samples with untreated polymer coating (**a**); and with polymer coating thermally treated at 430 °C (**b**).

Figure 4a shows that the untreated sample, sample set #1, has high selectivity for hydrogen gas. However, the typical trend in metal oxide-based sensors of increasing gas response with increasing OPT is not observed. The highest gas response was registered at an operating temperature of 250 °C, followed closely by the response at 350 °C, with 120% and 110%, respectively. One possible explanation could be that the polymer structure gradually changes with increasing operating temperature, resulting in a mixture of structural features that could lead to reduced gas permeability and partial blockage of the sensor layer. At even higher operating temperatures, the transformation from the ring to a cage-like structure within the polymer is potentially further advanced, leading to again more accessible gas pathways and higher gas responses. This structural transition could explain the observed gas response. However, this phenomenon requires further investigation.

Apart from hydrogen, other gas responses were also registered for 2-propanol, ethanol, n-butanol and carbon dioxide, with significantly lower gas responses, however. Also, it is important to mention that the complex response in Figure 4a is not a commonly met bell-shaped curve, as usually observed in previous research [48,49,50]. Considering that TiO_2_ nanostructures can be achieved in different crystalline states, such as anatase and rutile [63], so thermal annealing at 450 °C caused TiO_2_ to crystallize to anatase [64], which might be the cause between the difference in gas response, as at 610 °C TiO_2_ is transitioning to the rutile phase [64]. Therefore, TiO_2_ thermally annealed at 450 °C, in combination with the coated polymer, supposedly showed altered pathways between the gas and the transiting oxygen species [65] from 250 to 300 to 350 °C at the surface of the sensor structure.

Figure 4b, on the other hand, presents the gas responses of a polymer-coated sample that was thermally annealed at 430 °C prior to gas exposure. When comparing both samples, sets #1 and #2, at an operating temperature of 250 °C, it can be observed that hydrogen selectivity increased and response to the 2-propanol and carbon dioxide vanished, yet improvement in sensitivity was not attained. At a higher operating temperature of 350 °C, the annealed sample registered a significantly higher response to hydrogen gas (180%). This indicates that thermal treatment enhances both selectivity and sensitivity, particularly at elevated temperatures, making the sensor more effective for hydrogen gas detection under such conditions.

**Figure 5:**
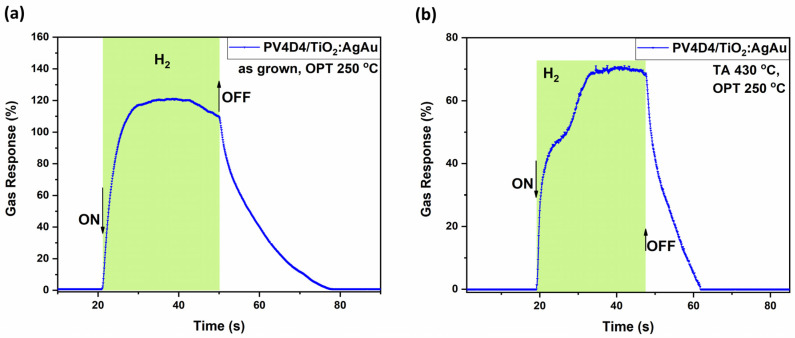
Dynamic response of the hybrid sensors based on TiO_2_ layers thermally annealed at 450 °C to hydrogen gas measured at an operating temperature of 250 °C with thermally treated and untreated polymer coatings on top. Samples with untreated polymer coating (**a**); and with polymer coating thermally treated at 430 °C, sample set #2 (**b**).

To better understand the key performance characteristics of this sample set, the highest gas responses from both annealed and as-deposited samples were selected for further analysis, focusing specifically on their dynamic response behavior. As shown in Figure 5a, for the sample with untreated polymer operated at 250 °C, a response of approximately 120% is registered. It is a stable response, with low saturation, a response time τ_res_ of ~5 s and recovery time τ_rec_ of ~20 s. On the other hand, Figure 5b shows the corresponding dynamic response of the annealed sample, which exhibits a lower maximal response, with a peak of 70%, yet demonstrates a faster recovery time (τ_rec_ ~ 10 s).

**Figure 6:**
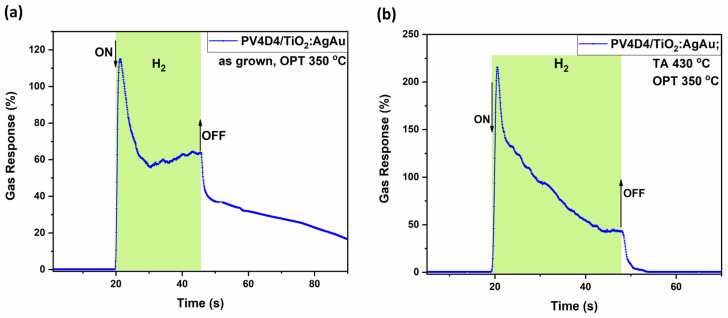
Dynamic response of the hybrid sensors based on TiO_2_ layers thermally annealed at 450 °C to hydrogen gas measured at an operating temperature of 350 °C with thermally treated and untreated polymer coatings. Samples with untreated polymer coating, #1, (**a**); and # 2 with polymer coating thermally treated at 430 °C (**b**).

As shown in Figure 6a, the sample set #1 with the untreated polymer operated at 430 °C registers a response of about 115% at its peak, while it slowly stabilizes itself at about 62%. It is a response with fast saturation, a response time of τ_res_ ~ 1.8 s and a recovery time τ_rec_ ~ of more than 40 s. On the other hand, the corresponding annealed sample set #2 (Figure 6b) shows a higher response, with a peak at 220%, yet faster saturation, stabilizing itself at about 48%. In comparison with Figure 5a, elevated operation temperatures lead to a decreased response time τ_res_ ~ 1.5 s and recovery time τ_rec_ ~ 1.8 s. This might occur due to the dependency of surface kinetics on operating temperature; thus, at higher operating temperature, the surface kinetics shows faster reactions [66,67].

**Figure 7:**
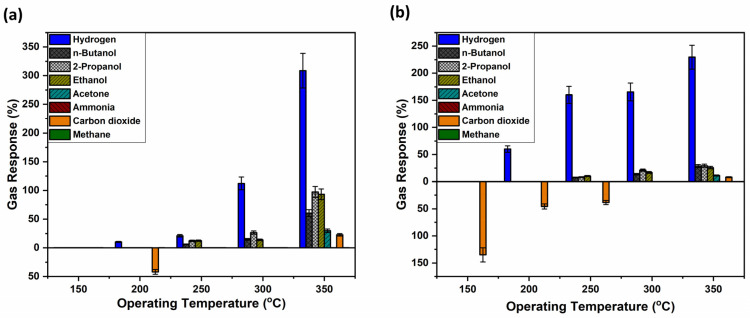
Gas responses of the hybrid sensor structures based on TiO_2_ films thermally annealed at 610 °C versus different gases and operation temperatures with thermally treated and untreated polymer coatings on top. Samples with untreated polymer coating (**a**); and with polymer coating thermally treated at 430 °C (**b**).

Figure 7 shows another set of samples, where this time 30 nm of TiO_2_ films, as the base of the sensor, has been thermally annealed at 610 °C in air, a temperature which showed the best performance in the treatment of TiO_2_ structures for higher gas response, as observed in our previous research works [47,49]. Gas responses have been plotted in both upward and downward directions along the y-axis to distinguish between different types of sensor behavior. The upward responses correspond to *n*-type behavior, which is characteristic of pristine TiO_2_-based sensors. In contrast, the downward responses indicate *p*-type behavior, which is attributed to the influence of Ag–Au bimetallic nanoparticles deposited on the surface of the sensor structure.

Figure 7a shows that annealing of the sensing structure at 610 °C leads to an increased selectivity for both hydrogen gas and carbon dioxide. However, carbon dioxide promotes a *p*-type behavior response at an operating temperature of 200 °C, with a gas response of 43%. The highest response to hydrogen gas was registered at an operating temperature of 350 °C, with a response value of 300%, with increased selectivity and sensitivity in comparison with other registered responses for butanol, 2-propanol, ethanol, acetone and carbon dioxide at the same temperature.

More data regarding this exclusive sample can be observed in Figure S1, where various tests at different relative humidity and concentrations have been made regarding the hydrogen response.

On the other hand, Figure 7b shows an even better selectivity for both hydrogen and carbon dioxide. Thermally annealing the polymer-coated sample provided a trade-off between decreased sensitivity and increased selectivity. Thus, although the highest response to hydrogen gas is measured at 350 °C, the value has slightly decreased to 230%. At other operating temperatures, however, the response to hydrogen gas increased compared to the untreated sample. Specifically, responses of 175% at 300 °C, 160% at 250 °C, and 60% at 150 °C were recorded.

Regarding carbon dioxide, its response behavior remained *p*-type for operating temperatures between 150 °C and 250 °C and shifted to *n*-type behavior at 350 °C. Other responses were 40% at 200 °C and 35% at 250 °C. As observed, *p*-type response behavior was registered exclusively for carbon dioxide, indicating high selectivity towards this gas. An interesting transition from *p*-type to *n*-type behavior is observed as the operating temperature increases, a phenomenon that requires further investigation. While TiO_2_ is considered an *n*-type semiconductor, recent studies, such as this proposed paper, report that it might exhibit both *n*- and *p*-type properties [68,69,70] while others have reported various materials having this transitory behavior [71,72]. Among the proposed reasons are either increasing its lattice oxygen activity or the incorporation of acceptor-type ions; in the case of Nowotny et al. [68], chromium was used, while in this study, Ag and Au bimetallic nanoparticles have been used.

At 150 °C, high selectivity for hydrogen gas was achieved; however, it was decided to investigate the dynamic response at 350 °C, as this was the best response.

**Figure 8:**
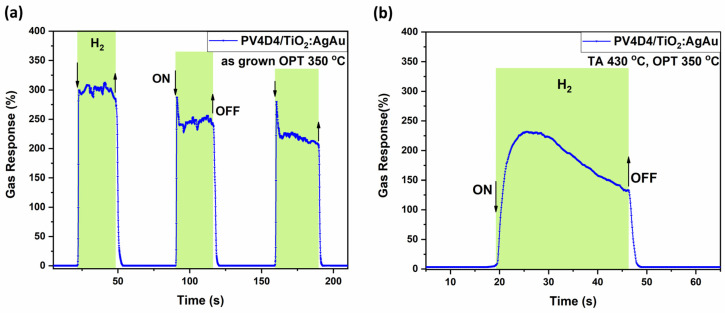
Dynamic response of the hybrid sensors based on TiO_2_ films thermally annealed at 610 °C to hydrogen gas at an operating temperature of 350 °C, with thermally treated and untreated polymer coatings on top. Sample sets with untreated polymer coating (**a**); and with polymer coating thermally treated at 430 °C (**b**).

Figure 8 presents the highest gas responses to hydrogen for both thermally treated and untreated polymer coating samples. Figure 8a shows the dynamic response to hydrogen gas of a hybrid sensor with an untreated polymer coating at an operating temperature of 350 °C with a response value of 300%, τ_res_ ~ 0.7 s and τ_rec_ ~ 1.5 s registered from first pulse, with bias voltage of 1.35 V from the source-meter. In Figure S3 in the Supplementary Materials, it can be observed that aging measurements eventually show a decrease in response after more than 900 days.

Figure 8b shows the dynamic response to hydrogen gas for thermally treated polymer coating samples atop the gas sensing structure at an operating temperature of 350 °C. The registered response is 230% with response and recovery times of τ_res_ ~ 4 s and τ_rec_ ~ 3 s, respectively. While the difference in response magnitude and dynamics is notable, the heat-treated sample offers improved selectivity towards hydrogen at elevated operating temperatures, making it a reasonable trade-off between sensitivity and selectivity.

**Figure 9:**
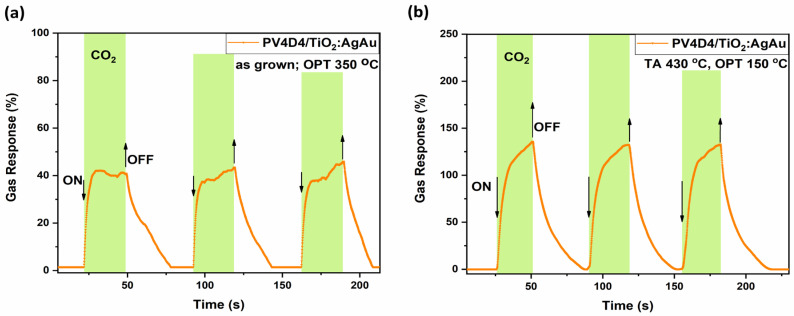
Dynamic response of the hybrid sensors based on TiO_2_ thermally annealed at 610 °C to carbon dioxide at different operating temperatures, with thermally treated and untreated polymer coatings atop. (**a**) Untreated polymer coating, OPT 200 °C; (**b**) polymer coating thermally treated at 430 °C, OPT 150 °C.

In Figure 9, the highest responses to carbon dioxide have been selected for both thermally treated and untreated polymer coating samples. Figure 9a shows the dynamic response to carbon dioxide of hybrid sensors with untreated polymer coating, with a response of 43%, τ_res_ ~ 1.4 s and τ_rec_ ~ 23 s registered from first pulse, at an operating temperature of 200 °C. Figure 9b shows the dynamic response to carbon dioxide for thermally treated polymer coating atop a gas sensing structure. The registered response is 130%, with τ_res_ ~ 3 s and τ_rec_ ~ 22 s.

While the difference is considerable, it can be considered an acceptable trade for securing increased selectivity towards carbon dioxide at relative higher operating temperatures from 150 to 250 °C. This shift, decrease in OPT from 200 °C (untreated polymer) to 150 °C (thermally treated polymer), is highly beneficial for reducing the power consumption of portable sensing devices.

## 4. Discussion

Understanding the significance of the obtained results is best achieved through comparison with the existing literature. Therefore, a comparative overview has been compiled and organized accordingly. In Table 1, various hydrogen gas sensors are presented, while in Table 2, different carbon dioxide sensors utilizing comparable tuning methods are listed, allowing for a broader evaluation of performance across different material systems and sensor architectures.

Table 3 provides a small comparison of different studies where Ag and Au nanoparticles have been used for gas sensing. This comparison shows that the intended results, such as increased selectivity, can be tuned by functionalization. Thus, in this work, Au nanoparticles have the purpose of increasing CO_2_ detection.

As observed, in the case of the proposed study, Ag and Au nanoparticles offer increased selectivity towards H_2_ and CO_2,_ while in other research, the main focus is on 2-propanol and n-butanol. Furthermore, it can be observed that there are faster response and recovery times compared to other papers, and in the case of CO_2_ detection, there is also a lower operating temperature.

Based on the data presented in this study and taking into account previously published results [49], a gas detection mechanism for the developed sensor structures is proposed. For H_2_, a similar detection mechanisms has been proposed in a previous paper [49], involving a *n*-type response with electrons (*e*^−^). The notable distinction in this work lies in the addition of Ag and Au nanoparticles on the surface of the TiO_2_ sensing layer and their influence on the detection mechanism.

A mechanism for CO_2_ gas detection was proposed, based on the *p*-type response behavior of the sensor, involving a positive hole (*p*^+^). According to a previous study [82], different concentrations of holes manifest a resistance change in the sensor. The following formulas are proposed:

(2) $$\frac{1}{2}{O_{2}}_{\underset{k_{-1}}{\leftarrow }}^{\overset{{k\;}_{1}}{\to }}O^{-}+p^{+}$$

In Equation (2), *k*_1_ is reducing carbon dioxide in carbon monoxide.

(3) $$p^{+}+O^{-}+CO\overset{k_{2}}{\to }{CO}_{2}$$

In Equation (3) *k*_2_ is forming carbon dioxide.

In case of the metal oxide-based sensor, it is known that there are several oxygen species at the surface of sensing structure, dependent on various operating temperatures, as stated in different papers [49,83,84,85]. In this paper, for CO_2_, the operating temperatures presented were 150 °C and 350 °C, as shown in Figure 9; therefore, the oxygen species at the surface are, respectively, 2O^−^ and O^2−^. Thus, the following equations are proposed as the gas sensing mechanism of CO_2_ for the proposed samples, according to this Equations (4)–(9) stated in [86].

(4) $${CO}_{2(gas)}+{OH}_{\left(adsorbed\right)}^{-}={HCO}_{3(surface)}^{-}$$

(5) $${CO}_{2(gas)}+H_{\left(adsorbed\right)}^{+}+{2e}_{\left(surface\right)}^{-}={HCO}_{2(surface)}^{-}$$

(6) $${HCO}_{2(surface)}^{-}=CO↑+{OH}_{\left(surface\right)}^{-}$$

(7) $${HCO}_{3(surface)}^{-}+{OH}_{\left(adsorbed\right)}^{-}={CO}_{3(bulk)}^{2-}+H_{2}O_{\left(gas\right)}$$

(8) $${CO}_{2(gas)}+O_{\left(adsorbed\right)}^{2-}={CO}_{3}^{2-}$$

(9) $$4{CO}_{2\left(gas\right)}+2\left(2O_{\left(adsorbed\right)}^{-}\right)={4CO}_{3}^{-}$$

Equation (8) represents the CO_2_ detection at 350 °C, while Equation (9) represents the detection at 150 °C. It is necessary to mention that the proposed mechanism is for gas molecules that pass through the polymer; therefore, it provides a rough idea for the adsorption process.

Taking into account the information published in a previous work regarding cyclo cage structures [47], as well as the current study, it can be concluded that the chosen annealing temperatures influence the degree of transition from a ring to a cage structure of the polymer. FTIR analysis indicates that annealing at 430 °C, as performed in this study, yields a lower distribution of cyclo cages relative to cyclo rings compared to the 450 °C used in previous studies.

This paper provides new insights into how polymer ultra-thin layers can be manipulated, thereby changing gas sensor properties. The novelty of this sensor is that it proposes new physical parameters for similar sensors based on TiO_2_; however, it has improved selectivity and sensitivity towards certain gases. In comparison with previous papers [47,48,49,50], in this work, the TiO_2_ base is thicker, with a rutile phase more sensitive to H_2_ detection, boosted with doped AgAu nanoparticles for CO_2_ detection. It was coated in PV4D4, which showed a different ratio of cyclo ring and cyclo cage pores, dependent on the thermal annealing of the polymer, for humidity resistance and better gas sensing properties. The ratio of ring and cage pores shows a severe impact on gas performance; as in previous work [47], the presence of a cage layer was found to influence selectivity and sensitivity, resulting in higher response levels towards hydrogen and acting as a molecular sieve for various gases. In contrast, this study shows that a lower distribution of cage structures within the polymer enables the tuning of selectivity towards CO_2_ with a *p*-type behavior response at 150 °C, while maintaining selectivity towards H_2_ for higher temperatures, ranging up to 350 °C.

Biomedical applications of this sensor include using it as an integrated component for capnometry. As mentioned in a recent article [87], the CO_2_ concentrations of one exhale is about 4% by volume, which corresponds to 40,000 ppm. Deviating data from this point could indicate either hypocapnia or hypercapnia in patients, caused by various factors such as panic attacks, respiratory failures, asthma, etc. [88]. Hydrogen, on the other hand, is found in lower concentrations. A recent study [89] showed there is a small variation in the H_2_ baseline range from 4 to 9 ppm regarding age, while it can be up to 20 ppm in the morning and 10 ppm later in the day. For the case of lactose intolerance, it is considered that an increase of 20 ppm above the base level shows a positive lactose test [90]. Another paper [91] showed that irritable bowel syndrome also shows an increase of 20 ppm above the baseline, as a result of fructose malabsorption.

## 5. Conclusions

This paper proposes a novel two-in-one sensor capable of detecting both hydrogen and carbon dioxide gas. Among the four investigated samples, the best-performing sensor was based on a TiO_2_ film, thermally annealed at 610 °C in air and functionalized with Ag Au bimetallic nanoparticles. The sensor was subsequently coated with a PV4D4 layer and annealed at 430 °C. For hydrogen gas, this sensor demonstrated *n*-type behavior, while for carbon dioxide, *p*-type behavior was observed. In addition, it showed a fast response and relatively fast recovery time, which was believed to be caused by a different ratio of cyclo cages and cyclo rings of PV4D4 formed on top of the sensor after thermal annealing.

It is strongly believed that precise thermal annealing of the PV4D4 in a specific temperature range allows for control of the ratio of cage to ring structures within the polymer. This enables tuning of the sensor’s selectivity and sensitivity through controlled changes in the sieve structures covering the underlying sensing layer. Furthermore, a decrease in operating temperature from 200 °C for the untreated polymer-coated sample to OPT of 150 °C for the thermally treated polymer-based sample set, is extremely beneficial for reducing the power consumption of portable sensing devices.

Due to these specific characteristics, it is possible to use the obtained sensor in environments where monitoring both of these gases is of interest. It can be even further tuned by simply thermally annealing at a certain temperature for a specific target gas.

The detection of hydrogen gas is important in the growing green energy industry and in biomedical diagnosis, as it has the potential to be a biomarker for various gastric diseases. Similarly, carbon dioxide is important as an industrial and medical gas used in various processes, as well as being a biomarker used in capnometry. Consequently, using one sensor type to measure both of these gases, as developed in this study, would be beneficial not only for the biomedical but also for the industrial sectors. Nevertheless, further investigation is required to better understand the underlying mechanisms and to determine its benefit for the detection of various targeted gases and their corresponding application niches. This type of two-in-one sensor opens up numerous possibilities for both research and practical use.

## Figures and Tables

**Table 1:** Hydrogen detectors tuned by nanoparticles NPs and polymer coatings on top.

No.	Sensors	NPs	Polymer	OPT, °C	Concentration, ppm	Response, %	Time, Response/Recovery, s
1.	Pd/PET [73]	Pd	PET polyethylene	RT	10,000	9	10/15
2.	PMMA/Pd NP/SLG hybrid sensor [74]	Pd	PMMA	-	20,000	65	1.81 min/5.52 min
3.	PV3D3/CuO/Cu_2_O/ZnO:Fe [51]	-	PV3D3	350	100	191	35/55
4.	Pd/PMMA/SiO_2_/Si [75]	Pd	PMMA	RT	600	1.6	5/6
5.	PV4D4/TiO_2_ [49]	-	PV4D4	300	100	100	
6.	PV4D4/TiO_2_:AgPt [47]	AgPt	PV4D4	350	100	709	3.02/23.23
7.	* PV4D4/TiO_2_:AgAu	AgAu	PV4D4	350	100	300	0.7/1.5
8.	** PV4D4/TiO_2_:AgAu	AgAu	PV4D4	350	100	230	4/3

*—this work, as-grown PV4D4. **—this work, thermally annealed PV4D4 at 430 °C.

**Table 2:** Carbon dioxide detectors tuned by nanoparticles NPs and polymer coatings.

No.	Sensors	NPs	Polymer	OPT, °C	Concentration, ppm	Response, %	Time, Response/Recovery, s
1.	Single-walled carbon nanotubes (SWCNTs)/ P(4VP−VBAz) [76]	-	P(4VP−VBAz)	RT	20,000	33	200/450
2.	Chem-FET/ P4VP-SWCNT [77]	Au	P4VP-SWCNT	RT	200	1	120/-
3.	cross-linked bacterial cellulose–amino graphene (CLBC-AmG)/ Polyaniline PANI [78]	-	Polyaniline PANI	RT	550	275	450/300
4.	Al_2_O_3_/Pt/ La_2_O_2_CO_3_ and P[VBTMA][PF_6_] [79]	-	La_2_O_2_CO_3_ and P[VBTMA][PF_6_]	RT	1000	11	-
5.	** PV4D4/TiO_2_: AgAu	AgAu	PV4D4	150	100	130	3/22
6.	** PV4D4/TiO_2_: AgAu	AgAu	PV4D4	250	100	43	1.4/23

**—this work, thermally annealed TiO_2_ films at 610 °C.

**Table 3:** Ag and Au NP influence on the performance of various metal oxides.

No.	Sensors	NPs	OPT,°C	Target Gas	Concentration, ppm	Response, %	Time, Response/Recovery, s
1.	(ZnO: Ag): AgAu [57]	AgAu bimetallic alloy	250	2-propnaol	100	156.5	25/23
2.	CuO/Cu_2_O: AgPt [80]	AgPt	275	2-propanol	100	275	20.4/75.2
3.	TiO2/CuO/Cu2O: AgPt [81]	AgPt	300	n-butanol	100	200	-
5.	** PV4D4/TiO_2_: AgAu	AgAu	350	H_2_	100	230	4/3
6.	** PV4D4/TiO_2_: AgAu	AgAu	150	CO_2_	100	130	1.4/23

**—this work, thermally annealed TiO_2_ films at 610 °C.

## Data Availability

The data presented in this study are available on request from the corresponding author.

## Supplementary Materials

The following supporting information can be downloaded at https://www.mdpi.com/article/10.3390/bios16010005/s1, Figure S1. Dynamic response of gas sensor to hydrogen of various concentrations (50, 100, 1000 ppm) at an operating temperature of 350 °C and to different relative humidity: (**a**) 17%; (**b**) 67%. Figure S2. Linear representation of Figure S1. Figure S3. Aging measurements of TiO2-based gas sensor, thermally annealed at 610° C, doped with AgAu nanoparticles and coated with a thin layer of PV4D4.
