# Supplementary material for: Two-in-One Hybrid Sensor Based on PV4D4/AgAu/TiO2 Structure for Carbon Dioxide and Hydrogen Gas Detection in Biomedical and Industrial Fields
Source: Biosensors (Basel). 2025 Dec 22;16(1):5. doi: 10.3390/bios16010005 (PMC12838770; doi:10.3390/bios16010005)
Supplement: Supplementary file 1 [file biosensors-16-00005-s001.zip › biosensors-3974143-supplementary.pdf]

biosensors

Supporting information

Two-in-One Hybrid Sensor Based on PV4D4/AgAu/TiO_2 Structure for Carbon Dioxide and Hydrogen Gas Detection in Biomedical and Industrial Fields

In supporting information, you can find **Figure S1, S2** and **S3**.

Figure S1 shows a comparison of hydrogen gas sensitivity at various concentrations for two different relative humidity both at operating temperature of 350° C. In **Figure S1 (a)**, relative humidity was ~17%, thus the response to 50, 100 and 1000 ppm were respectively 15%, 32% and 182%. Registered response times were ~2s while recovery time was 4, 2.5 and 8 seconds. Figure S1 (b) shows responses at the same concentration for a higher relative humidity of ~67%. The registered responses were 12%, 24% and 260%, while response time were registered ~4s, ~2.8s and ~16s to peak reach and recovery times respectively ~5s, ~10s and ~8s.

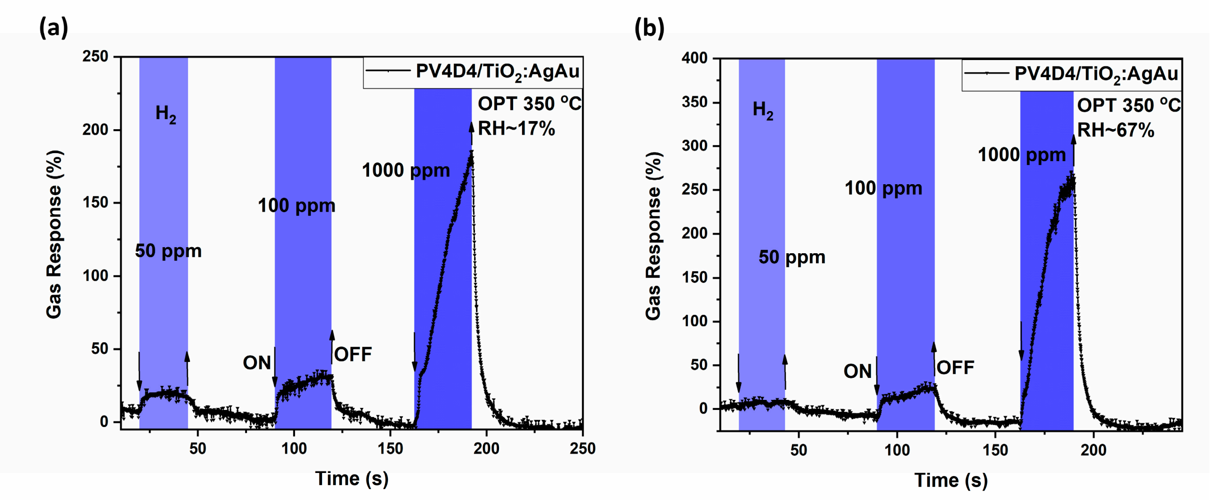

Figure S1. Dynamic response of gas sensor to hydrogen of various concentration (50, 100, 1000 ppm) at operating temperature of 350 °C to different relative humidity: **a)** 17%; and **b)** 67%.

Figure S2 shows a linear graph for comparison of H_2 response at OPT of 350 °C for ~17% and ~67% of relative humidity at 50, 100 and 1000 ppm

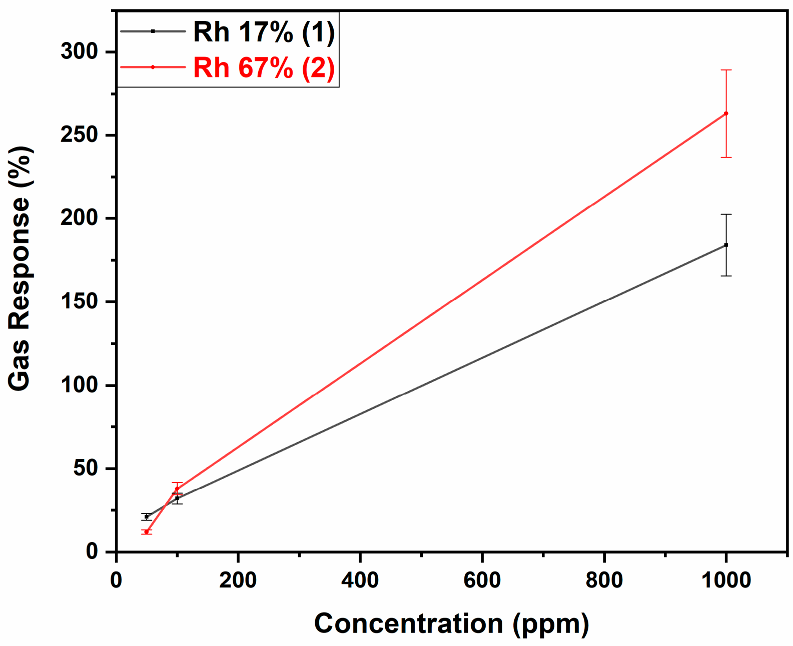

Figure S2. Linear representation of Figure S1.

Figure S3 show H_2 response at different points of time, starting with 0 as initial measurement and followed by 90, 181, 364, 730 and 921 days after first measurement.

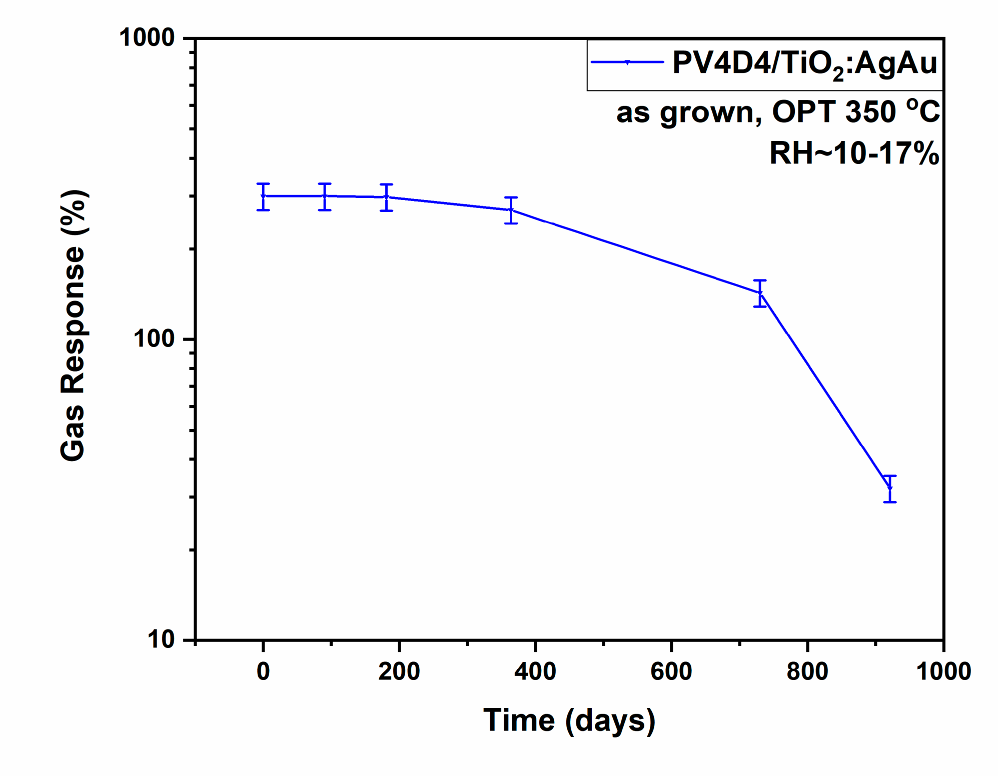

Figure S3. Aging measurements of TiO_2 based gas sensor, thermally annealed at 610° C, doped with AgAu bimetallic nanoparticles and coated with a thin layer of PV4D4.

Conclusions. Thermal annealing of pV4D4 allows for structural tuning of its sieve-like properties, enhancing gas sensor selectivity and sensitivity while reducing operating temperatures to 150 °C for more energy-efficient detection.
